# Could Nanotheranostics be the Answer to the Coronavirus Crisis?

**DOI:** 10.1002/gch2.202000112

**Published:** 2021-03-24

**Authors:** Dina A. Mosselhy, Mhd Adel Assad, Tarja Sironen, Mady Elbahri

**Affiliations:** ^1^ Nanochemistry and Nanoengineering Department of Chemistry and Materials Science School of Chemical Engineering Aalto University Espoo 02150 Finland; ^2^ Department of Virology Faculty of Medicine University of Helsinki P.O. Box 21 Helsinki 00014 Finland; ^3^ Department of Veterinary Biosciences Faculty of Veterinary Medicine University of Helsinki P.O. Box 66 Helsinki 00014 Finland; ^4^ Microbiological Unit Fish Diseases Department Animal Health Research Institute Dokki Giza 12618 Egypt; ^5^ Nanochemistry and Nanoengineering Institute for Materials Science Faculty of Engineering Kiel University 24143 Kiel Germany; ^6^ Center for Nanotechnology Zewail City of Science and Technology Sheikh Zayed District Giza 12588 Egypt

**Keywords:** anti‐coronavirus, appraisals, coronavirus, electrospun facemasks, nanodiagnostics, nanoparticles

## Abstract

The COVID‐19 pandemic is expanding worldwide. This pandemic associated with COVID‐19 placed the spotlight on how bacterial (e.g., methicillin‐resistant *Staphylococcus aureus*) co‐infections may impact responses to coronavirus. In this review the ways in which nanoparticles can contain and rapidly diagnose COVID‐19 under the umbrella of nanotheranostics (i.e., smart, single agents combining nanodiagnostics and nanotherapeutics) are elaborated. The present work provides new insights into the promising incorporation of antiviral nanotheranostics into nanostructured materials, including electrospun fibers with tailored pore sizes and hydrophobicity, namely “superhydrophobic self‐disinfecting electrospun facemasks/fabrics (SSEF).” SSEFs are proposed as smart alternatives to address the drawbacks of N95 respirators. The challenges of coronavirus containment are underscored, literature is reviewed, and “top‐five suggestions” for containing COVID‐19 are offered, including: i) preventive appraisals—avoiding needless hospital admission and practicing frequent hand washing (from 20 to 60 s). ii) Diagnostics—highly recommending nanodiagnostics, detecting COVID‐19 within 10 min. iii) Therapeutics—expanding nanotherapeutics to treat COVID‐19 and bacterial co‐infections after safety assessments and clinical trials. iv) Multipronged and multinational, including China, collaborative appraisals. v) Humanitarian compassion to traverse this pandemic in a united way.

## Graphical abstract

Antiviral nanotheranostics, including plasmonic nanoparticles (NPs), quantum dots, and polymeric NPs could concurrently diagnose and treat coronaviruses. Nanodiagnostics rapidly detect coronaviruses via color change, fluorescence, and electrical response. Nanotherapeutics combat coronaviruses via preventing infection initiation and viral replication, and inhibiting endosomal acidification. Electrospun nanofibers, namely “Superhydrophobic Self‐disinfecting Electrospun Facemasks/Fabrics” could traverse N95 respirators’ drawbacks.

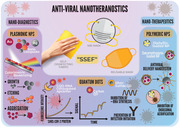

## 1 Introduction

The coronavirus pandemic (coronavirus disease 2019, COVID‐19) induced by severe acute respiratory syndrome coronavirus 2 (SARS‐CoV‐2) plagues the world. To question the race of researchers to develop vaccines against SARS‐CoV‐2 and the measures adopted by different governments in response to the COVID‐19, we should understand the science of the coronavirus. We need to think outside the box for novel diagnostic and therapeutic strategies combating COVID‐19. For example, could nanoparticles (NPs) or nanotheranostics play a dual role in defeating this pandemic? We have also to highlight overlooked areas by exploring the details pertaining to coronavirus infections. What could be the burden of methicillin‐resistant *S. aureus* (i.e., MRSA) in co‐infections with coronavirus?

Coronaviruses (CoVs) are enveloped and positive single‐stranded RNA (the genome size is around 26 to 32 kilobases as the largest genome for RNA viruses) viruses within the family Coronaviridae. The term “coronavirus” describes the appearance of CoV virions under the electron microscope with the spikes radiating from the viral membrane forming a crown, which is “corona” in Latin. CoVs share key features, such as their genome encode 16 nonstructural proteins followed by structural proteins (spike, envelope, membrane, and nucleocapsid). CoVs comprise four genera, namely alpha‐CoV (group 1), beta‐CoV (group 2), gamma‐CoV (group 3), and delta‐CoV (group 4). The infectious human CoV (HCoV) strains were thought to be only six, namely HCoV‐229E (229E), HCoV‐OC43 (OC43), SARS‐CoV, HCoV‐NL63 (NL63), HCoV‐HKU1 (HKU1), and Middle East respiratory syndrome coronavirus (MERS‐CoV). Where, 229E, OC43, and NL63 are spread globally.^[^ ^1^ ^]^ Previous research among patients suffering acute respiratory tract infections of HCoV over seven years in Guangzhou, China, demonstrated a seasonal prevalence of 229E, NL63, and OC43 to peak during spring and autumn. The symptoms of such HCoV infections swayed from common cold‐like symptoms to influenza‐like symptoms, asthma, and until pneumonia.^[^ ^2^ ^]^

In 2002 and 2003, a global outbreak of SARS was reported and related to first exposures to a single ill health care worker from Guangdong Province, China, visiting Hong Kong. He was hospitalized with SARS and died. Invaluable collaborative epidemiologic investigation of nineteen clinical specimens from patients in seven countries (Vietnam, Singapore, Thailand, Hong Kong, Canada, Taiwan, and the USA) was delivered to the Centers for Disease Control and Prevention. The authors have isolated a novel coronavirus from patients, meeting the SARS criteria, of 80 to 140 nm size surrounded by 20 to 40 nm complex surface projections. Sequencing of the viral genomes from 12 of these patients showed the isolate to be a novel coronavirus distantly related to previously sequenced CoVs, namely SARS‐associated coronavirus. Even though the origin of this novel SARS‐associated coronavirus remains elusive, the authors assumed that the virus originated in animals, then mutated, causing person‐to‐person transmission.^[^ ^3^ ^]^ Li and colleagues^[^ ^4^ ^]^ have consolidated the link between bats and CoVs by reporting that horseshoe bats are a natural reservoir of CoVs, namely SARS‐like coronaviruses (SL‐CoVs), tightly linked to SARS‐CoV (human strains) and both nestle phylogenetically within the same spectrum. What jumps out in the reported results is the 92% identical nucleotide sequence between SL‐CoV Rp3 (PCR product of the fecal bat sample from Hubei) and SARS‐CoV Tor2 (strain from a patient in Toronto).^[^ ^5^ ^]^ The authors have finally rung a worrying alarm about the genetic diversity of zoonotic viruses in bats and the possibility to cross the threshold of the species barrier, causing future human outbreaks, specifically in places including human–animal interaction (e.g., wet‐market systems).

In the period between September 2012 and January 20, 2017, the World Health Organization (WHO) received reports from 27 countries of 1879 human patients infected with the MERS‐CoV resulting in 659 deaths. Dromedary camels are the most probable source of animal‐to‐human transmission. MERS‐CoV initiates infection of bronchial epithelial cells via binding to dipeptidyl peptidase 4 (DPP‐4) receptor and spreads, entering the lung parenchymal cells. MERS‐CoV replicates within the cytoplasm of the host cells.^[^ ^6^ ^]^ Previous work on the epidemiology and pathogenesis of HCoV has established the importance of learning the lessons from SARS‐CoV and MERS‐CoV outbreaks to be prepared with a powerful arsenal against the next CoV outbreak.^[^ ^1^ ^]^ In December 2019, however, patients with exacerbated pneumonic conditions of an unknown etiological agent were linked to the seafood and wet market in Wuhan, China. Sequencing of the RNA genome extracted from the bronchoalveolar‐lavage fluid of patients matched the genome from lineage B of the genus betacoronavirus with a nucleotide sequence that was 86.9% identical with an earlier published genome of bat SARS‐like CoV (bat‐SL‐CoVZC45, MG772933.1). The 2019‐nCoV is a novel beta‐CoV causing novel coronavirus‐infected pneumonia and different from SARS‐CoV and MERS‐CoV. The 2019‐nCoV particles are spherical (with little pleomorphism), about 60 to 140 nm in size, and possess characteristic spikes (9 to 12 nm), creating the crown shape surrounding the virions.^[^ ^7^ ^]^ Governments should alleviate human panic from the COVID‐19 pandemic by funding the scientific communities to understand the science of SARS‐CoV‐2, which is dynamic with ongoing advancements in research.^[^ ^8^ ^]^ What have we learned about COVID‐19 in light of the pandemic events? One study by Chen and coworkers^[^ ^9^ ^]^ reported that of the 99 SARS‐CoV‐2 infected patients in Wuhan, 67 were men, and 32 were women. The main symptoms were fever or cough, and a third of patients had shortness of breath. Muscle ache, headache, confusion, chest pain, and diarrhea were also reported. Half of the infected patients had an underlying chronic disease (i.e., cardiovascular and cerebrovascular diseases and diabetes). The first point of interest in this study is the sex bias of SARS‐CoV‐2, infecting more males than females most probably due to the protection of X chromosome and female sex hormones in the heightened innate and adaptive immunity. Sex bias of the SARS‐CoV‐2 was also reported in an earlier SARS‐CoV study, where infected male mice were more susceptible than the same‐age female infected mice. This bias was attributed to the role of female estrogen signaling in suppressing SARS‐CoV replication, together with the protective X chromosome and female sex hormones.^[^ ^10^ ^]^

Research has also revealed the hospital‐associated transmission of COVID‐19.^[^ ^11^ ^]^ Among the 138 hospitalized confirmed COVID‐19 cases in Zhongnan Hospital, Wuhan, a cluster of healthcare workers (40, 29%) mainly in the same department, and patients hospitalized for another underlying cause (17, 12.3%) in the same wards, contracted the infection. These incidences constituted 41.3% percentage of hospital‐associated COVID‐19 infections. Other research^[^ ^12^ ^]^ has reported a high incidence of SARS‐CoV‐2 infection with age (over 60 years) and concurrent cancer (non‐small cell lung carcinoma) due to compromised immunity. The infection rate of cancer patients with SARS‐CoV‐2 (0.79%) in Zhongnan Hospital, Wuhan, was higher than the cumulative incidence of all confirmed cases in the city of Wuhan over the same period (0.37%), leading to the conclusion that hospital admission and visits are risk factors for contracting SARS‐CoV‐2 infection. Taking a closer look at case fatality rates, in China, the exploratory analysis of the first 72 314 confirmed cases of COVID‐19 revealed that the age group of ≥80 years recorded the highest case fatality rates, reaching 14.8%.^[^ ^13^ ^]^ In the US (data from 49 states), the case fatality rates proportionally increased with the increase in age. No deaths were reported among persons of ≤19 years, but the highest case‐fatalities (10% to 27%) were reported among older adults aged ≥85 years.^[^ ^14^ ^]^ A recent work^[^ ^15^ ^]^ shed light on the observed new multisystem inflammatory syndrome (MIS‐C, including cytokine storm) in SARS‐CoV‐2 infected school‐aged children. The work compared the observed clinical MIS‐C features with Kawasaki disease (KD, children's disease, including coronary arteries dilatation). Interestingly, several features were different, including first, epidemiological bias with MIS‐C correlation to African origin children, while no cases were detected in China and Japan. Second, children age bias with SARS‐CoV‐2, MIS‐C correlated to older children and adolescents, while KD observed in children <5 years old.^[^ ^15^ ^]^

Secondary bacterial infections (i.e., *Acinetobacter baumannii*, *Klebsiella pneumoniae*, and *Aspergillus flavus*) are common in the severely ill patients, which aggravates the COVID‐19 condition due to the virulence of pathogens and the overwhelmed immunity of the patient. Co‐bacterial infection is critical, resulting in the death of one COVID‐19 patient out of the 29 isolated confirmed patients suffering from fever associated pneumonia in Tongji hospital (Huazhong University of Science and Technology, Wuhan, China) in January 2020.^[^ ^16^ ^]^ Most studies on the COVID‐19 pandemic have overlooked the critical issue of co‐bacterial infections, however. Besides, lessons from the past have taught us that the resistant MRSA producing Panton–Valentine leukocidin toxins have been associated with invasive pneumonia and bad prognosis,^[^ ^17^, ^18^ ^]^ which could aggravate the severity of COVID‐19. A recent paper delineating the therapeutic and triage regimes of coronavirus cases in clinics^[^ ^19^ ^]^ reported that patients positive for COVID‐19 with compromised systemic and local respiratory defenses suffer from bacterial co‐infection. The authors suggest a measure four regime. This regime comprises oxygen supply, isolation‐ward admission, arbidol (antiviral) treatment, and nemonoxacin or linezolid (antibiotics treating *Streptococcus pneumoniae* and MRSA) administration. This antibiotic treatment is highly important as co‐infections exaggerate the severity of COVID‐19 illness.^[^ ^20^ ^]^ Considering also the global bacterial antibiotic resistance crisis and the cries for novel antimicrobials, therefore, extending the demanded antimicrobial agents to defeat the COVID‐19 crisis and other viral co‐infections (e.g., influenza viruses) would be invaluable.

**Figure 1:**
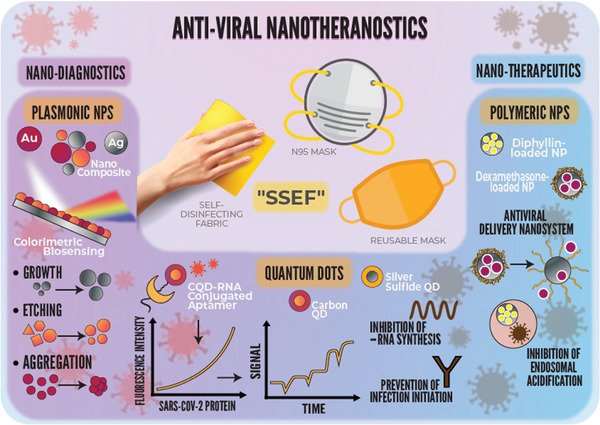
A schematic diagram explaining the science behind the concurrent diagnosis and treatment modalities of the severe acute respiratory syndrome coronavirus 2 (SARS‐CoV‐2) using different antiviral nanotheranostic systems, namely, plasmonic NPs, QDs, and polymeric NPs. Antiviral nanotheranostics simultaneously diagnose (i.e., via colorimetric biosensing, fluorescence signaling, and electrical response signals) and treat (i.e., via prevention of infection initiation, prevention of viral replication and budding, and inhibition of endosomal acidification). The promising application of the antiviral nanotheranostics would be incorporating smart electrospun facemasks and self‐disinfecting fabrics, which we propose as “Superhydrophobic Self‐disinfecting Electrospun Facemasks/Fabrics, SSEF.”

On August 2, the worldwide COVID‐19 pandemic reached almost 17 million confirmed infections and 700 000 deaths,^[^ ^21^ ^]^ and on December 21, 2020, the Public Health England^[^ ^22^ ^]^ declared several 23 mutations (i.e., 13‐non synonymous mutations, 4 amino acid deletions, and 6‐synonymous mutations) in the new SARS‐CoV‐2 variant, VUI‐202012/01 (“Variant Under Investigation”). Within October and November 2020, another South African SARS‐CoV‐2 variant with critical mutations in the receptor‐binding domain circulated widely in Eastern and Western Cape Provinces.^[^ ^23^ ^]^ But why are nanotheranostics still not considered to be a stellar “out‐of‐the‐box” solution? First, nanotheranostics could be defined as a combined single dance (single agent) of therapeutics and diagnostics using the nanotechnology platform. Although the theranostic definition dates back to two decades ago, nanotheranostics could generally contribute to personalized or precision medicine,^[^ ^24^ ^]^ and more specifically to COVID‐19 via the rapid‐diagnosis and detailed therapeutic mechanisms proposed in this work. Rapid diagnosis would be valuable in the global war against COVID‐19 because early‐stage diagnosis facilitates infected patients’ rapid isolation. Intermediate‐stage diagnosis and late‐stage diagnoses facilitate proper treatment and discharge of patients, respectively.^[^ ^25^ ^]^ **Figure** **1** provides an overview of how nanotheranostics, mainly plasmonic NPs, quantum dots (QDs), polymeric NPs (as antiviral delivery nanosystems), and their combinations could simultaneously diagnose and treat COVID‐19 when applied in superhydrophobic self‐disinfecting electrospun facemasks/fabrics (SSEF). Plasmonic NPs, such as Au NPs, Ag NPs, and their composites, have played critical roles as disease biomarkers in colorimetric biosensing assays because of their unique optical properties, primarily localized surface plasmon resonance (LSPR). LSPR is a phenomenon generated by the interaction of incident light with the NP (smaller than the incident wavelength) metal surfaces, causing the free electrons’ oscillation in a coherent localized plasmon oscillation with resonant frequencies. These frequencies are highly dependent on the size, morphology, composition, orientation, and interparticle distances of plasmonic nanostructures.^[^ ^26^, ^27^ ^]^ Anisotropic plasmonic nanostructures engender higher sensitivity in LSPR properties than their isotropic counterparts. This higher sensitivity is highly appreciated in the biosensing of ultralow analyte concentrations.^[^ ^26^ ^]^ Huang and coworkers^[^ ^28^ ^]^ developed a rapid (i.e., within 15 min), sensitive (i.e., limit of detection of 307 pseudovirus particles/mL), and optical measurement of SARS‐CoV‐2 virus particles using a spike protein‐specific nanoplasmonic resonance sensor integrated with a chip cartridge (Figure 1). Colorimetric sensing is invaluable for bio‐applications requiring rapid and onsite detection (e.g., the current COVID‐19 pandemic). The mechanisms governing the colorimetric biosensing using plasmonic nanostructures originated from the tailoring of size and morphology, composition, orientation, and distances between NPs. Consequently, the biosensing modalities could be grouped as belonging to i) bioanalyte‐induced aggregation and assembly, ii) etching, and iii) growth and transformation.^[^ ^26^ ^]^ Moitra et al.^[^ ^29^ ^]^ developed a colorimetric assay for naked‐eye detection of SARS‐CoV‐2 based on the change in the surface plasmon resonance of the capped Au NPs (with oligonucleotides specific for SARS‐CoV‐2 N gene) after agglomeration (Figure 1). Recently, considerable literature has dealt with the theme of carbon quantum dots (CQDs)‐based biosensors. CQDs are evolving fluorescent‐carbon nanostructures (of size <10 nm and structure of carbon core and surface oxygen‐containing functional groups) full of promise in precise biosensing applications. CQDs demonstrate normal, down‐converted, or up‐converted (of better biocompatibility and specific benefit in photodynamic therapeutic applications) photoluminescence. CQDs are also easily synthesized and functionalized. The fluorescence mechanisms of CDS evolved from i) the bandgap transitions facilitating the fluorescence by conjugated π‐domains, and ii) surface defects facilitating the fluorescence emission. The different working mechanisms of CQD‐based biosensors are fluorescence quenching (attributed to the charge transfer), static quenching, dynamic quenching, energy transfer, inner filter effect, photo‐induced electron transfer, and fluorescence resonance energy transfer (attributed to the energy transfer from a high‐energy fluorescent donor to a low‐energy, boosting the fluorescence).^[^ ^30^ ^]^ Recently, an authoritative review summing up the NP‐based SARS‐CoV‐2 diagnostics referred to the ideal SARS‐CoV‐2 detection role of QDs in studying the S protein‐acetylcholine angiotensin‐converting enzyme 2 binding dynamics and internalization due to their small size, photostability, and the ease of their surface functionalization with biological molecules.^[^ ^31^ ^]^

On the question of antiviral properties, functionalized Ag NPs,^[^ ^32^ ^]^ Au NPs (and with similar virus dimensions),^[^ ^33^, ^34^ ^]^ and carbon nanodots^[^ ^35^ ^]^ and porous silicon NPs have been used as antiviral agents.^[^ ^36^ ^]^ Moreover, our previous studies have demonstrated the competent antibacterial efficacy of Ag NPS (<10 nm) in defeating *S. aureus* solely^[^ ^37^ ^]^ or beating MRSA in a composite with silica.^[^ ^38^ ^]^ Several studies showed anti‐coronavirus NP properties, including Łoczechin et al.,^[^ ^39^ ^]^ that showed carbon QDs inhibition of human coronavirus (HCoV) receptors (DPP‐4), blocking the initiation of viral infection (Figure 1). Du et al.^[^ ^40^ ^]^ demonstrated the inhibition of Ag^2^S QDs to the porcine epidemic diarrhea virus (i.e., a CoV model) negative‐strand RNA synthesis (Figure 1). The in‐depth mechanistic details governing the anti‐CoV properties are displayed below (i.e., Section 2.2. Nanotherapeutics Encompass Anti‐CoV Actions).

Unfortunately, there is a gap between treatment strategies of infectious diseases and the scientific understanding of the mechanisms that trigger surviving such infections, for example, COVID‐19 survival.^[^ ^41^ ^]^ Therefore, this manuscript makes several significant contributions to unveiling the scientific facts of COVID‐19. The present study explores the critical roles played by different antiviral nanotheranostics in diagnosing and curbing coronavirus infections while shedding light on the different NP‐mediated mechanisms of actions and potential applications, as shown in Figure 1. We provide essential insights into the challenges hindering the advancement of antiviral nanotheranostics and pandemic containment. We close by highlighting our critical opinions, spotting the research gaps, and providing outstanding solutions for defeating COVID‐19.

## 2 Antiviral Nanotheranostics

### 2.1 Nanodiagnostics for CoV

**Figure 2:**
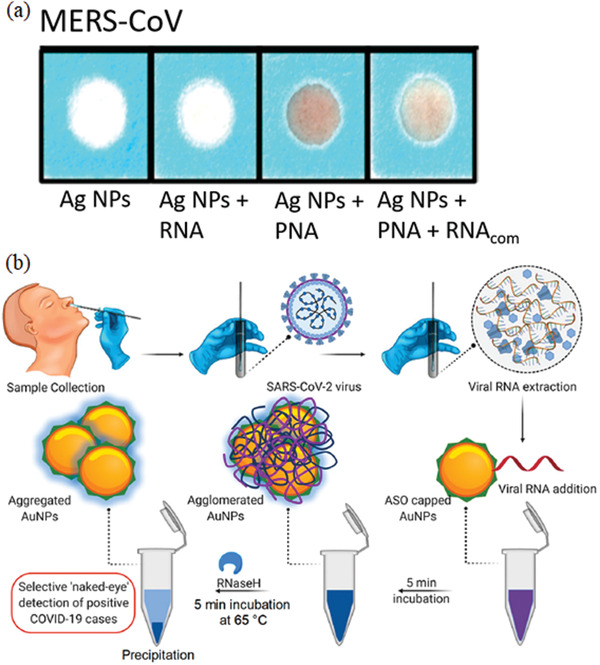
Nanodiagnostics of Middle East respiratory syndrome coronavirus (MERS‐CoV). Selective colorimetric detection of MERS‐CoV using a paper‐based colorimetric RNA sensor in the presence of complementary RNA (RNAcom). a) The pyrrolidinyl peptide nucleic acid (PNA) probe hybridized the viral RNA, forming a duplex dispersed Ag NPs and produced a detectable changed color. Reproduced with permission.^[^ ^42^ ^]^ Copyright, 2017, American Chemical Society. b) A schematic illustration of the ten min‐selective naked‐eye (color change from violet to dark blue) detection of SARS‐CoV‐2 RNA facilitated the agglomerated Au NPs capped by thiol‐modified antisense oligonucleotides (ASO) specific for N‐gene (nucleocapsid phosphoprotein) of viral RNA. Reproduced with permission.^[^ ^29^ ^]^ Copyright, 2020, American Chemical Society (ACS).

The diagnosis of COVID‐19 is a critical requirement in managing the pandemic. At the beginning of the epidemic, clinicians diagnosed positive patients using conventional nucleic‐acid detection kits. However, such kits are time‐consuming.^[^ ^25^ ^]^ Teengam and colleagues^[^ ^42^ ^]^ have improved MERS‐CoV detection by developing a paper‐based colorimetric RNA sensor. The sensor relied on a positively charged pyrrolidinyl peptide nucleic acid (acpcPNA) probe aggregating the negatively charged Ag NPs in the absence of complementary RNA. While in the presence of MERS‐CoV RNA, the acpcPNA probe hybridized the viral RNA, forming a negatively charged RNA‐acpcPNA duplex. The duplex dispersed the Ag NPs because of the electrostatic repulsion and produced a detectable color change. This paper‐based colorimetric RNA sensor [as depicted in **Figure** **2**a] provided a diagnostic benchmark for an easy, rapid (within 10 min) and low detection limit of MERS‐CoV with no necessity of multiple PCR cycles. Moitra and colleagues^[^ ^29^ ^]^ have developed another 10 min‐colorimetric assay for rapid and selective naked‐eye detection of SARS‐CoV‐2 (i.e., a detection limit of 0.18 ng µL^−1^ of RNA), without any expensive, sophisticated instruments, after the change in the surface plasmon resonance (i.e., a redshift of ≈40 nm) of the capped Au NPs (size of 55 nm). This change (i.e., redshift with a color change from violet to dark blue) stemmed from the agglomeration of Au NPs capped with thiol‐modified antisense oligonucleotides specific for two regions of the SARS‐CoV‐2 N gene (nucleocapsid phosphoprotein gene) in the presence of its targeted SARS‐CoV‐2 RNA sequence. Besides, the added RNaseH cleaved the RNA strand from the RNA‐DNA hybrid, producing a visually (naked‐eye) detectable precipitate facilitated by the additional Au NPs’ aggregation [as delineated in Figure 2b]. Kim and collaborators^[^ ^43^ ^]^ have developed an easy, rapid colorimetric assay for onsite MERS‐CoV detection. The assay involved the hybridization of designed thiol‐modified probes with the targeted complementary bases of MERS‐CoV RNA, forming disulfide bonds that induced a self‐assembled complex. The complex protected the Au NPs from salt‐induced aggregation and limited the color change for diagnosis of MERS‐CoV. The spectral analysis of the colorimetric assay showed that in the absence of the target, the wavelength was redder shifted compared with the spectrum in the target's presence.

Roh and Jo^[^ ^44^ ^]^ designed another easy and rapid diagnostic tool for SARS‐CoV that utilized a QDs‐conjugated RNA aptamer chip for specific and sensitive detection of nucleoside (N) protein of SARS‐CoV that is a principal viral pathological agent in infected hosts. The chip had a coupling coating allowing the efficient immobilization of captured SARS‐CoV N protein. The selective N protein detection was shown as high fluorescence signals on the chip under the confocal microscope (at a low concentration of 0.1 pg mL^−1^). In contrast, no fluorescence signal was found for bovine serum albumin. The authors suggested the fitness of their technique to detect multiple infectious agents. Considering biosensors, Seo and coworkers^[^ ^45^ ^]^ fabricated a field‐effect transistor (FET) for onsite, specific SARS‐CoV‐2 detection in clinical specimens. The COVID‐19 sensor was designed by conjugating the SARS‐CoV‐2 spike antibody onto the FET's sensing graphene sheets via a linker, namely 1‐pyrenebutyric acid N‐hydroxysuccinimide ester. It detected the SARS‐CoV‐2 spike protein at a starting concentration of 100 fg mL^−1^ in the clinical transport medium. The SARS‐CoV‐2 detection stemmed from the changes in the graphene channel's surface potential and the corresponding electrical response signals. The sensor SARS‐CoV‐2 limit of detection was recorded as 2.42 × 10^2^ copies/mL in nasopharyngeal swabs of COVID‐19 patients, implying the high sensitivity of the fabricated COVID‐19 FET sensor with no need for sample pretreatment.

### 2.2 Nanotherapeutics Encompass Anti‐CoV Actions

**Figure 3:**
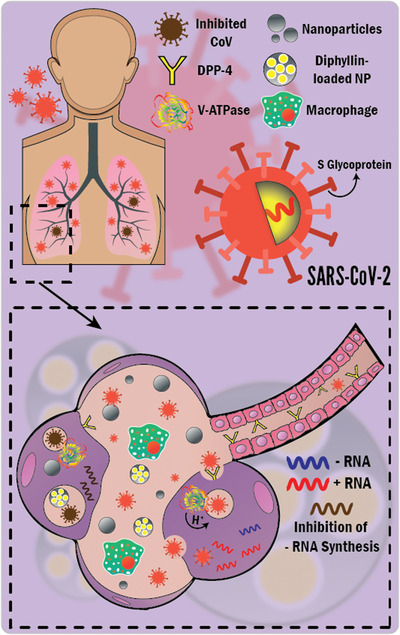
Therapeutic anti‐CoV mechanisms of NP gunshots. The mechanisms include several inhibitions. 1) Inhibition of the binding of CoV spike (S) glycoprotein to DPP‐4 receptors, preventing initiation of infection. 2) Inhibition of the synthesis of viral negative‐strand RNA (ribonucleic acid), preventing viral replication and budding. 3) Inhibition of endosomal acidification by diphyllin [V‐ATPase inhibitor]‐loaded NP shots preventing viral infection cascade and stalling CoV within the endosome.

The most interesting question of this manuscript is how NPs could play a substantial role as anti‐CoV agents? The answer to this question is expressed in **Figure** **3**.

Few studies have dealt with the antiviral effects and mechanisms of NP shots against CoVs. Łoczechin and coworkers^[^ ^39^ ^]^ showed the antiviral properties of carbon QDs (8 nm) against HCoV with a half‐maximal effective concentration as low as 5 µg mL^−1^. They have described the antiviral mechanisms as the interaction of carbon QDs with the S1 subunit of the spike (S) glycoprotein of the virus, inhibiting the binding of HCoV to the host receptors (DPP‐4), blocking the initiation of infection and subsequent viral entry into the host cells and the inhibition of viral RNA replication. On the same token, Ting and collaborators^[^ ^46^ ^]^ prepared curcumin‐cationic carbon dots (1.5 nm) with antiviral properties against the porcine epidemic diarrhea virus as a coronavirus model. The reported anti‐coronavirus mechanisms were i) changing the structure of surface proteins; ii) the positively charged dots electrostatically interact with the negatively charged virus, resulting in aggregation of the virus and reduction of its infectivity; and iii) inhibition of viral replication and budding. Du and colleagues^[^ ^40^ ^]^ have identified the therapeutic antiviral effects of spherical Ag_2_S nanoclusters (QDs) with average diameters of 3 and 4 nm against porcine epidemic diarrhea virus, inhibiting the synthesis of viral negative‐strand RNA and viral budding. They added that the main ruinous antiviral effects were not attributed to Ag^+^ ions. Hu and collaborators^[^ ^47^ ^]^ encapsulated diphyllin within poly(ethylene glycol)‐block‐poly(lactide‐co‐glycolide) NPs of an average diameter of 40 nm and showed the enhanced antiviral effects of the loaded‐polymeric NPs against feline coronavirus (FCoV). Endosomal acidification allows the viral infection cascade (viral uncoating and cytoplasmic entry to the host cells), where vacuolar ATPase (V‐ATPase) pumps protons into the endosomal vesicles. Diphyllin (a V‐ATPase inhibitor) inhibited endosomal acidification in infected cells, inhibiting viral replication. The polymeric NPs improved the safety profile and antiviral inhibitory effects of diphyllin, increasing its therapeutic index by 800‐fold against FCoV. In the same context of polymeric NPs, we propose dexamethasone encapsulation (Dex, i.e., corticosteroid) in such antiviral nanodelivery systems because it has been described as a breakthrough, reducing hospitalized ventilated COVID‐19 patient deaths by one third.^[^ ^48^ ^]^ Furthermore, previous studies showed a more remarkable, targeted reduction of inflammatory cytokines and recovery in comparison with pristine Dex^[^ ^49^ ^]^ or when synergistically combined with naproxen (i.e., nonsteroidal anti‐inflammatory drug (NSAID)).^[^ ^50^ ^]^

### 2.3 Electrospun Nanofibers for Theranostic Masks and Self‐Disinfecting Fabrics

**Figure 4:**
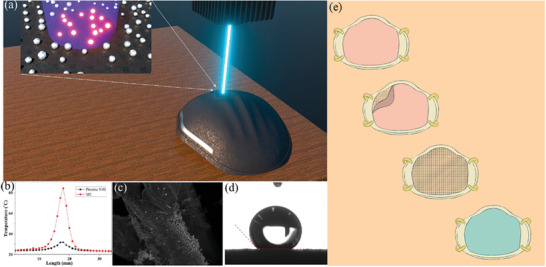
a) A schematic Illustration of the laser diode (405 nm) decontamination. b) The plasmonic heating of the deposited silver nanoparticles (Ag NPs) increased the surface temperature of the M2 sample (i.e., method 2 including a second step of transforming the deposited Ag NPs onto polyimide film via continuous wave mode laser‐induced forward transfer) up to 80 °C in comparison with the pristine N95 respirator. c) Field emission scanning electron microscope image and d) contact angle measurement demonstrating the superhydrophobic properties of M2 sample after laser disinfection (100 cycles). Panels (a to d) are reproduced with permission.^[^ ^58^ ^]^ Copyright, 2020, American Chemical Society. e) A schematic illustration presents nanoporous membrane placement on an 8 in silicon‐on‐insulator wafer onto a reusable N95 respirator after its folding. Panel (e) is reproduced with permission.^[^ ^61^ ^]^ Copyright, 2020, American Chemical Society (ACS).

**Figure 5:**
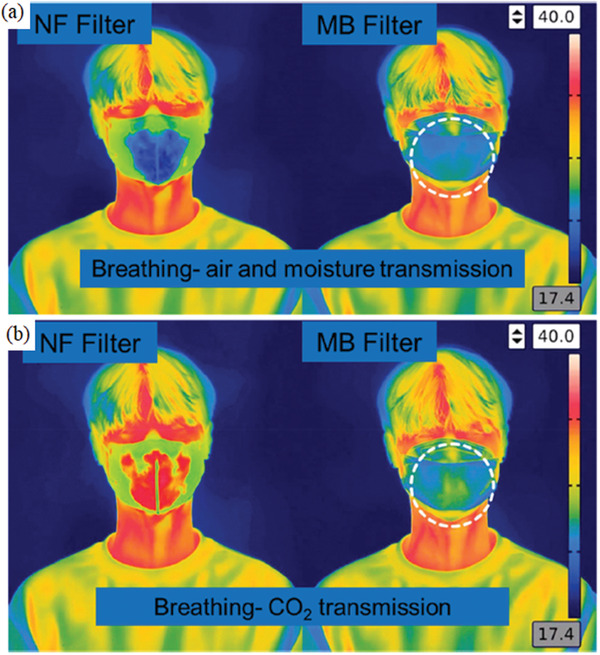
Assessment of the breathability captured by a thermal infrared camera, demonstrating more efficient a) air and moisture transmission and b) CO_2_ transmission b) via NF filters than MB filters. Reproduced with permission.^[^ ^57^ ^]^ Copyright, 2020, American Chemical Society (ACS).

Regrettably, airborne transmission of aerosols shed from asymptomatic individuals (i.e., silent shedders) during breathing and speaking contributes mostly to the spread of SARS‐CoV‐2. Such aerosols could result in air infectivity indoors for hours and be deeply inhaled into the lungs, making universal masking a priority to mitigate this aerosol transmission. This masking is highly demanded in places favor the accumulation of high viral concentrations, including hospital settings, flights, restaurants, and crowded places suffering limited ventilation.^[^ ^51^ ^]^ However, most of the respiratory masks possess a larger pore size than the SARS‐CoV‐2 size (around 120 nm). Electrospinning is a simple, straightforward, and economical process for producing nanofibers (NF).^[^ ^52^, ^53^, ^54^, ^55^ ^]^ Electrospun filter membranes hold tremendous potential for filtration via tailoring their unique properties (e.g., uniform morphology and high porosity and surface areas).^[^ ^56^, ^57^ ^]^ Electrospun fibers could participate efficiently in the world‐war fighting the COVID‐19 pandemic. During the delay time until the development of the SARS‐CoV‐2 vaccine, the manufacturing of a massive number of respirators and masks is genuinely needed to avoid the respiration of infectious aerosols and to mitigate the bleeding expenses of using N95 respirators. Despite the high‐level protection provided by N95 respirators, they present three drawbacks. First, N95 respirators lack self‐disinfecting properties.^[^ ^58^ ^]^ Second, N95 facemasks are typically made of melt‐blown (MB) respirator filters^[^ ^57^ ^]^ and are recruited for single‐use.^[^ ^59^ ^]^ Third, their filtration efficiency remains around 85% for particles <300 nm because of its wider pore size (≈300 nm).^[^ ^60^ ^]^ Zhong and colleagues^[^ ^58^ ^]^ addressed the first self‐disinfecting challenge via generating a plasmonic photothermal, self‐disinfecting, and superhydrophobic coating on N95 respirators. The plasmonic heating of the deposited Ag NPs (with sizes of 20 to 50 nm, where the size increased with the thickness increase of the precursor Ag films) increased the surface temperature up to 80 °C within only 1 min of sunlight illumination. The released Ag^+^ ions from the Ag NPs play the disinfection role, and the superhydrophobic properties (also engendered from the graphene included in the composite) hinder the accumulation of respiration droplets on the respirator surfaces [as seen in **Figure** **4**a–d]. To improve the filtration efficiency of N95 respirators, El‐Atab and colleagues^[^ ^61^ ^]^ have developed a Si‐based nanoporous (5 nm) template (i.e., through a combination of patterning and potassium hydroxide etching of a silicon‐on‐insulator wafer). The template could then be used as a hard mask, transferring the patterns onto a lightweight (<0.12 g) hydrophobic anti‐fouling polymeric membrane. The produced membranes could then be placed onto reusable N95 respirators, preventing SARS‐CoV‐2 infections [as illustrated in Figure 4e]. Airflow rates were theoretically calculated, achieving >85 L min^−1^ via the mask that ensured its breathability. Ullah and collaborators^[^ ^57^ ^]^ have compared the reusing efficiency of polypropylene MB filter (as representative of N95 respirator) with an electrospun polyvinylidene difluoride‐based NF filter after ethanol (75%) treatment including, spraying (up to ten cycles per each three times press) and dipping (from 5 min and up to 24 h). The investigated filter performance parameters after treatment were as follows i) filtration efficiency that was maintained high in NF filters, while it dropped in MB filters to ≈62% and 65% after 10 cycles and 24 h ethanol treatment, respectively, pointing toward the unsuitability of reusing MB filters. ii) The airflow rate reached ≈27.2 cfm in the MB filter before and after treatment and 17 cfm in the NF filter before treatment that decreased slightly after treatment. The authors speculated that the lower air permeability of the NF filter after treatment was attributed to the partial depolymerization of the spun‐bond polyethylene terephthalate, decreasing pore sizes. iii) Breathability (influenced by the water vapor transport rate, WVTR) was superior in the NF filter than the MB filter (as presented in **Figure** **5**) due to the finer structure and uniform morphology and pore sizes of NF filters, allowing the transmission of water vapors. The sponge‐like structure of MB filter also aided in resisting moisture transmission. iv) The final parameter was reserved for investigating the biocompatibility of filters on human keratinocyte (HaCaT) and endothelial HUVEC, demonstrating the absence of any cytotoxicity of NF filter and slight toxicity of the MB filter toward HUVEC cells.

Konda and coworkers^[^ ^60^ ^]^ demonstrated that the aerosol (aerosol‐based viral transmission) filtration efficiencies of multiple fabric layers (i.e., combinations of cotton‐silk, cotton‐chiffon, and cotton‐flannel) were enhanced, reaching >80% (for particles <300 nm), when compared with the performance of single fabric layers (5 to 80% for particles <300 nm). They linked the combinations’ enhanced filtration efficiencies to the synergistic effects of physical and electrostatic‐based filtrations. They proposed a need for the future development of cloth masks, considering the leakage challenges (i.e., from the gaps between mask ends and facial contours), efficient air exhalation (i.e., after crossing the humidity challenge triggered by exhalation), and the criteria of repetitive use and washing. Leung and Sun^[^ ^62^ ^]^ simulated SARS‐CoV‐2 and its attached aerosol by sodium chloride aerosols (50 to 500 nm) and tested electrospun PVDF NFs (of diameters 84, 191, 349, and 525 nm that were further electrostatically charged by corona discharge) for filtration efficiency. They achieved ultrahigh filtration efficiency up to 94% and low‐pressure drop of 26 Pa by using a multimodal approach combining large‐diameter NFs (525 nm) with large basis weight (6 L, 0.765 gsm). Furthermore, incorporation of active compounds (e.g., tartaric acid and lupeol) from Indian medicinal plants with potential anti‐CoV properties into respiratory masks through the electrospinning process could reinforce their physical barrier potential with anti‐CoV potential. These plant‐incorporated electrospun masks would provide several benefits in this pandemic battlefield, such as i) increasing the breathability of masks because of the porosity of electrospun fibers; ii) decreasing the manufacturing expenses of respiratory masks; iii) supplying masks to both the healthcare workers and the ordinary people.^[^ ^63^ ^]^ Our explicit recommendation is the functionalization of electrospun NF filters (i.e., of tailored pore sizes, uniform morphology, high surface areas, and superhydrophobic properties) with antiviral nanotheranostic systems translating our message into “Superhydrophobic Self‐disinfecting Electrospun Facemasks/Fabrics, SSEF”.

## 3 Challenges Hindering Advancement and Pandemic Containment

Even with the inspiring properties of plasmonic NPs in the colorimetric biosensing field, this field is limited by the complicated synthesis routes of anisotropic plasmonic NPs, other than Au NP and Ag NPs, and their low production yield. Thus, there is a definite need for more standardized, straightforward protocols with higher yields for anisotropic plasmonic NPs.^[^ ^26^ ^]^ Though aptamers, as a viral detection technology, could equip the world in its war against viral pandemics, only a few aptamer‐based products are commercially available. Furthermore, screening of aptamers is usually executed under specific conditions that could not precisely mimic the intricate conditions of clinical samples. Therefore, the structure, function, and binding properties of aptamers could substantially change in clinical samples. Adding to that, the costs of specific bases required for constructing aptamers with high sensitivity and specificity constitute more obstacles facing the flourishing of the aptamer viral detection technologies.^[^ ^64^ ^]^ Despite considerable efforts in reporting and sequencing CoVs, significant problems would continue if the reservoir host distribution and transmission routes of SARS‐CoV are not known, making it more problematic to prevent and contain future SARS outbreaks.^[^ ^4^ ^]^ To date, no single filter standard technology exists for capturing airborne CoVs. Although electrospun NF filters could be tailored to capture airborne CoVs ranging in size from 60 to 140 nm^[^ ^62^ ^]^ and demonstrate superior filtration efficiency, breathability, and cytocompatibility performances than MB filters, the reusability remains a challenge.^[^ ^57^ ^]^ Therefore, nanotheranostic systems could provide stellar solutions, serving the dual roles of diagnostics and self‐disinfections using electrospun NF filters.

A deeply saddening situation was the limited donations received after the request of the Director General (Tedros Adhanom Ghebreyesus) on January 2020, for generous donations of 675 million USD. This requested donation was intended to reduce the healthcare strain of the COVID‐19 pandemic in developing countries. Only 54·5 million USD were received before the appalling collapse of worldwide stock markets.^[^ ^8^ ^]^ The COVID‐19 crisis was abetted by misleading drug advice (i.e., claiming that anti‐inflammatory drugs, such as ibuprofen, could exacerbate the infection) that was not supported by substantial scientific evidence. Therefore, patients in chronic pain receiving NSAIDs should not quit taking them to avoid the advent of COVID‐19 and should avoid shifting to opiates. Bearing in mind that critically ill patients with SARS‐CoV‐2 infections should not also receive NSAIDs as there is no scientific evidence for the advantage.^[^ ^65^ ^]^ We believe that using re‐purposed drugs or new antiviral drugs to treat COVID‐19 without proper clinical trials could also inflame the pandemic crisis. The emergence of rumors could also exacerbate the chaotic situation surrounding the pandemic crisis. Such rumors included the use of Chinese traditional medicinal plants, saunas, drinking high‐alcohol liquor, and smoking could be used for the prevention and treatment of COVID‐19 without any scientific evidence.^[^ ^66^ ^]^

## 4 Our Critical Opinions: What Stellar Answers?

Hospital settings may host the spread of SARS‐CoV‐2 (vide supra), therefore contracting SARS‐VoV‐2 infections could be prevented by avoiding needless hospital visits and practicing frequent hand washings. Hand hygiene has shown to be effective in preventing SARS‐CoV‐2 and MRSA co‐infections. Hand hygiene is best performed for 20 to 30 s (if hands are not visibly dirty) and 40 to 60 s (if hands are visibly dirty) using soap and water. Since NPs possess antiviral properties against CoVs. Therefore, we also advise considering nanotheranostics and their combinations with antiviral drugs for development, safety evaluation, clinical trials, and approval as stellar theranostic agents combating COVID‐19.

As early as January 2020, researchers^[^ ^67^ ^]^ emphasized the importance of vigilant control measures to contain the COVID‐19 epidemic in China. Now, with the COVID‐19 infections in pandemic proportions, the media could deliver invaluable messages (e.g., hand washing, avoiding “unprotected” contact with live animals, avoiding travel, staying home, isolation of suspected individuals, and seeking providers or emergency care) to prevent the spread of COVID‐19.^[^ ^13^, ^14^, ^68^ ^]^ Social distancing is recommended among different aged‐persons to slow the viral spread, reduce the stress on healthcare systems, and protect our beloved and most vulnerable older adults.^[^ ^14^ ^]^

Researchers have urged the scientists working within multidisciplinary sectors of chemistry, life sciences, and medicine to develop technologies that allow rapid, accurate, and safe diagnosis of COVID‐19.^[^ ^25^ ^]^ This rapid diagnosis is highly needed to provide accurate information for healthcare providers, making patient‐appropriate management decisions. Therefore, creative thinking of novel and rapid diagnostics to be used in public health laboratories and clinical laboratories is highly regarded.^[^ ^69^ ^]^ Although the currently used nucleic acid amplification‐based diagnostics (e.g., real‐time reverse transcription‐PCR) for SARS‐CoV‐2 are reliable, they remain laborious, require a long time could reach a couple of days, and need to be performed in a diagnostic laboratory. Therefore, we heartily recommend the incorporation of nanodiagnostics that could enable fast (within a few minutes) and onsite detection of SARS‐CoV‐2 in the developed technologies for further approval. Despite the high sensitivity and fast diagnostic ability of the colorimetric biosensing assays based on the anisotropic plasmonic nanostructures, the varied ability of color recognition among laypeople and light‐sources errors remain a concern. Therefore, color‐picker software and smartphone‐applications could provide concrete solutions to the color recognition challenge,^[^ ^26^ ^]^ paving the way for smart digital and artificial intelligence applications of the developed antiviral nanotheranostics. Furthermore, Au NPs used by the Moitra^[^ ^29^ ^]^ and Kim^[^ ^43^ ^]^ groups for the colorimetric detection of SAS‐CoV‐2 and MERS‐CoV were spherical NPs. Therefore, we propose the administration of other nanostructures of Au nanomaterials (rather than the spherical Au NPs) in the developed antiviral nanotheranostic systems. This proposal stems from the much higher sensitivity of Au nanorods, nanoplates, nanoflowers, nanoframes, nanocubes, and nanobipyramids to refractive index changes than low‐dimensional spherical Au NPs.^[^ ^26^ ^]^ Regarding therapy, chloroquine phosphate (discovered in 1934) is a cheap and safe antimalarial drug that helped as a drug revival to efficiently allay the COVID‐19 associated pneumonia in clinical trials in China.^[^ ^70^ ^]^ Chloroquine executes its anti‐coronavirus effects via increasing the endosomal pH. This increase inhibits the viral infection cascade (e.g., cleavage of spike glycoprotein by endosomal cathepsins to allow fusion of viral envelope and host endosomal membrane) that necessitates endosomal acidification,^[^ ^71^ ^]^ and captivates the virus within the endosome. Furthermore, a consequence of the possible falling of synthetic NPs within the same nanometer size scale of SARS‐CoV‐2 is a possible extra anti‐SARS‐CoV‐2 mechanism of chloroquine. This extra mechanism includes the inhibition of clathrin‐mediated endocytosis of nano‐sized particles due to suppression of phosphatidylinositol binding clathrin assembly protein (regulating membrane curvature and endocytosis).^[^ ^72^ ^]^ However, skepticisms regarding the efficacy of chloroquine against SARS‐CoV‐2 have been raised by several scientists, referring to the absence of successful chloroquine treatment of acute viral infection in animals or humans.^[^ ^73^ ^]^ This skepticism of chloroquine exceeded far beyond the absence of efficacy against SARS‐CoV‐2 to becoming harmful, impacting the host immune response to SARS‐CoV‐2 negatively.^[^ ^73^ ^]^ Following genome sequencing of SARS‐CoV‐2, scientists tirelessly worked on developing promising vaccines, and the Coalition for Epidemic Preparedness Innovations is on the threshold of clinically trying eight vaccines. Once any of the vaccines show safety and efficacy in animal models could be directly tried on a larger scale (that was thought to be around June 2020)^[^ ^74^ ^]^ and has currently come to reality. The fact that influenza vaccination reduces pneumonia development chances raised an assumption that it could also have a side‐service of preventing secondary MRSA infections.^[^ ^20^ ^]^

We believe that electrospinning could play a critical role in covering the current market shortage of protective gear (i.e., masks) in the time of coronavirus crisis, especially for the frontline healthcare workers and after creatively crossing the electrospinning challenges (i.e., their biocompatibility that remains a concern because of the possible presence of impurities (e.g., residual solvents and linkers), triggering immune responses.^[^ ^54^ ^]^ Researchers could play with the pore size (while considering the breathability of filters) and hydrophobicity of electrospun fibers to prevent the penetration of SARS‐CoV‐2 and fouling of filters, respectively. Functionalizing electrospun NF filters with antiviral nanotheranostic systems would simultaneously self‐disinfect the filters and alarmingly change their color or signal to ultimately meet the safety requirements of the market.

## 5 Summary and Outlook

This manuscript has reviewed the COVID‐19 pandemic and its bacterial co‐infections. We proposed several antiviral nanotheranostic systems in this work, delineating the detailed anti‐CoV mechanisms of nanotheranostics that facilitate competent therapy and rapid diagnosis of COVID‐19. We provided smart alternatives for the disadvantages of N95 respirators via incorporating antiviral nanotheranostics into electrospun fibers with tailored pore sizes, termed “Superhydrophobic Self‐disinfecting Electrospun Facemasks/Fabrics, SSEF.” We have reflected critically and stimulated debate on the investigated literature in this review manuscript and offered promising appraisals for COVID‐19 containment. Our “top‐five suggestions” are i) preventive, for example, stopping unnecessary hospital visits and hand hygiene (from 20 to 60 s) and universal masking, especially in places with limited ventilation. ii) Diagnostic measures include highly recommended incorporation of nanodiagnostics for onsite detection of SARS‐CoV‐2 for further approval. iii) Therapeutics, such as, considering NPs in combinations with antiviral drugs for treating COVID‐19 and its co‐bacterial infections, especially in SEFF. iv) Multipronged collaborations between multidisciplinary teams and countries. v) Humanitarian actions include compassionate treatment to safely cross the COVID‐19 crisis. What is now recommended is a cross‐national study involving the implementation of these appraisals.

Our future work is directed to review the biocompatibility of such promising antiviral nanotheranostics and answers critical safety and other essential looming outstanding questions. Namely, what is the pressing need and priority in combating COVID‐19? For example, should we focus on developing antiviral theranostic strategies, or developing disease‐tolerance drugs that enhance patients’ survivability, or a long‐lived married strategy combining theranostics and disease‐tolerance drugs is the current priority? Would combinations of antiviral nanotheranostics with antibiotics hit COVID‐19 and resistant co‐bacterial infections hard? Would nanotheransotics be safe when applied in facemasks?

## Conflict of Interest

The authors declare no conflict of interest.

## Authors Contributions

D.A.M. and M.E. designed the study and collected authoritative scientific research articles and data. D.A.M. mainly wrote the manuscript and critically interpreted the reviewed literature. D.A.M. drew the preliminary sketches of the original Figures. M.A.A. mainly constructed all the original and reproduced Figures. All the authors revised the manuscript with renowned inputs provided by T.S.
